# Concurrent suppression of Aβ aggregation and NLRP3 inflammasome activation for treating Alzheimer's disease†

**DOI:** 10.1039/d1sc06071f

**Published:** 2022-02-21

**Authors:** Tao Yang, Lei Zhang, Yicun Shang, Zhenzhu Zhu, Suxing Jin, Zijian Guo, Xiaoyong Wang

**Affiliations:** State Key Laboratory of Pharmaceutical Biotechnology, School of Life Sciences, Nanjing University Nanjing 210023 P. R. China boxwxy@nju.edu.cn; School of Food Science and Pharmaceutical Engineering, Nanjing Normal University Nanjing 210023 P. R. China jinsuxing@njnu.edu.cn +86 25 89684549 +86 25 89684549; State Key Laboratory of Coordination Chemistry, School of Chemistry and Chemical Engineering, Nanjing University Nanjing 210023 P. R. China

## Abstract

Alzheimer's disease (AD) is a neurodegenerative illness accompanied by severe memory loss, cognitive disorders and impaired behavioral ability. Amyloid β-peptide (Aβ) aggregation and nucleotide-binding oligomerization domain (NOD)-like receptor protein 3 (NLRP3) inflammasome play crucial roles in the pathogenesis of AD. Aβ plaques not only induce oxidative stress and impair neurons, but also activate the NLRP3 inflammasome, which releases inflammatory cytokine IL-1β to trigger neuroinflammation. A bifunctional molecule, 2-[2-(benzo[*d*]thiazol-2-yl)phenylamino]benzoic acid (BPBA), with both Aβ-targeting and inflammasome-inhibiting capabilities was designed and synthesized. BPBA inhibited self- and Cu^2+^- or Zn^2+^-induced Aβ aggregation, disaggregated the already formed Aβ aggregates, and reduced the neurotoxicity of Aβ aggregates; it also inhibited the activation of the NLRP3 inflammasome and reduced the release of IL-1β *in vitro* and *vivo*. Moreover, BPBA decreased the production of reactive oxygen species (ROS) and alleviated Aβ-induced paralysis in transgenic *C. elegans* with the human Aβ_42_ gene. BPBA exerts an anti-AD effect mainly through dissolving Aβ aggregates and inhibiting NLRP3 inflammasome activation synergistically.

## Abstract 2

Bifunctional molecule BPBA inhibits Aβ aggregation and NLRP3 inflammasome activation, thereby decreasing ROS and IL-1β *in vitro* and *vivo*; it synergistically prevents Alzheimer's disease *via* alleviating Aβ neurotoxicity and reducing neuroinflammation.

## Graphical abstract

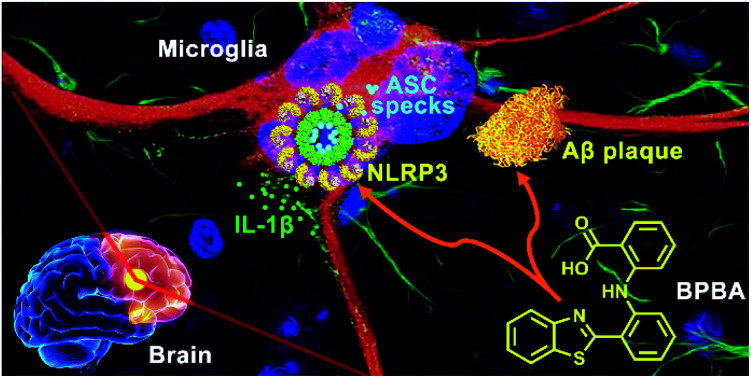

## 1. Introduction

Alzheimer's disease (AD) is a common form of dementia characterized by the accumulation of extracellular amyloid β-peptide (Aβ) plaques, neuroinflammation and neuronal cell death in the brain.^1^ About 50 million people are living with AD globally in 2019, which put an enormous economic and mental burden on the society and families.^2^ Although great effort has been made, the pathogenesis and pathogenic factors of AD are not yet fully elucidated.^3^ Existing anti-AD drugs merely delay the symptoms to some extent but cannot stop the progression of the disease and have various side effects.^4^ The Aβ cascade hypothesis is the most prevalent supposition about the pathogenesis of AD, which suggests that Aβ deposits play a vital role in initiating the disease.^5,6^ In the past few decades numerous studies focused on cellular Aβ deposits as a pathological hallmark and target of therapeutic drugs.^7^ The newly FDA approved aducanumab is the first anti-AD drug based on the Aβ cascade hypothesis, though its efficacy is inconclusive.^8^

Recently, increasing evidence supported that innate immunity-mediated neuroinflammation plays a crucial role in the pathogenesis and progression of AD.^9^ Inflammasome plays an important role in neuroinflammation and neurodegenerative diseases.^10,11^ Particularly, the microglia-specific nucleotide-binding oligomerization domain (NOD)-like receptor protein 3 (NLRP3) inflammasome mediates the pathogenesis of AD.^12,13^ The NLRP3 inflammasome is an intracellular multimeric protein complex composed of the receptor protein NLRP3, the effector protein cysteine protease-1 (caspase-1), and the adaptor protein called apoptosis-associated speck-like protein containing a caspase activation and recruitment domain (ASC).^14^ It affects a variety of physiological functions, including the innate immune process and caspase-1-dependent response.^15,16^ Aβ plaques activate the NLRP3 inflammasome, which releases inflammatory factors such as cytokines interleukin-1β (IL-1β), interleukin-18 (IL-18), and ASC.^17^ The released inflammatory cytokines and ASC trigger chronic neuroinflammation and lead to cognitive impairment.^12,18^ In reverse, neuroinflammation aggravates the formation of Aβ fibers and plaques, and worse, boosts tau phosphorylation, thus leading to their aggregation to promote the pathogenesis of AD.^16^ The activation of the NLRP3 inflammasome/caspase-1 axis contributes much to AD *in vivo*,^10^ while the deficiency of NLRP3 or caspase-1 markedly reduces the Aβ burden and cognitive impairment in amyloid precursor protein/presenilin-1 (APP/PS1) mice.^19^ The elevation of IL-1β in the brain has been associated with the progression and onset of AD.^20,21^ The inhibition of IL-1β could significantly diminish brain nerve inflammation, alleviate cognitive impairment, and partially reduce Aβ deposition in 3xTg-AD mice.^22^

Various inhibitors of the inflammasome have been reported,^23^ such as OLT1177,^19,24^ CY-09,^25^ tranilast,^26^ oridonin,^27^ benzenesulfonamide analogues,^28^ sulphonamides (CRIDI, MCC950),^29–31^ and non-steroidal anti-inflammatory drugs (NSAIDs).^12,32^ More inhibitors are targeted to Aβ aggregation;^33–35^ however, inhibitors that emphasize both Aβ aggregation and inflammasome are rare.

**Fig. 1:**
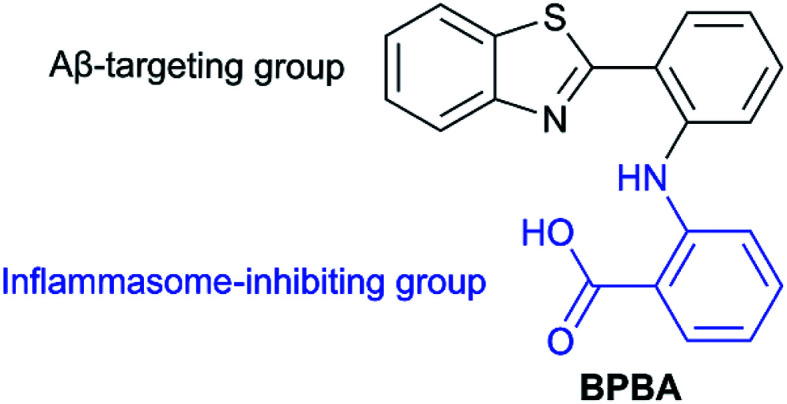
Chemical structure of BPBA.

Since the synergism between Aβ oligomers or plaques and pro-inflammatory factors could increase the neural damage to the brain,^17^ a combination therapy involving the inhibition of Aβ aggregation and NLRP3 inflammasome activation may enhance the therapeutic effect on AD. Herein, we integrate benzothiazole, an Aβ-targeting group, with *o*-aminobenzoic acid, an analogue of the NLRP3 inflammasome inhibitor mefenamic acid,^32^ into a single molecule BPBA (Fig. 1), which may lead a dual inhibition of NLRP3 inflammasome activation and Aβ aggregation simultaneously. A series of experiments demonstrate that BPBA remarkably inhibits the self- and metal-induced Aβ aggregation, reduces the level of inflammatory cytokine IL-1β, restrains the activation of caspase-1 *in vitro*, and alleviates the formation of Aβ oligomers and plaques as well as the Aβ-associated toxicity *in vivo*.

## 2. Results and discussion

### 2.1. Design, synthesis and physicochemical properties

The design of BPBA is based on the structures of benzothiazole and *o*-aminobenzoic acid; the former is a potential Aβ-targeting group that has a specific affinity for Aβ aggregates rich in β-sheet structures, and the latter is an analogue of mefenamic acid, which is a known inhibitor of the NLRP3 inflammasome.^32^ In addition, the O, N, and S atoms in BPBA could chelate metal ions, which may inhibit the metal-induced Aβ aggregation. We suppose that BPBA has the capability to prevent the formation of Aβ plaques and restrain the activation of the NLRP3 inflammasome in the brain of AD sufferers.

**Scheme 1:**
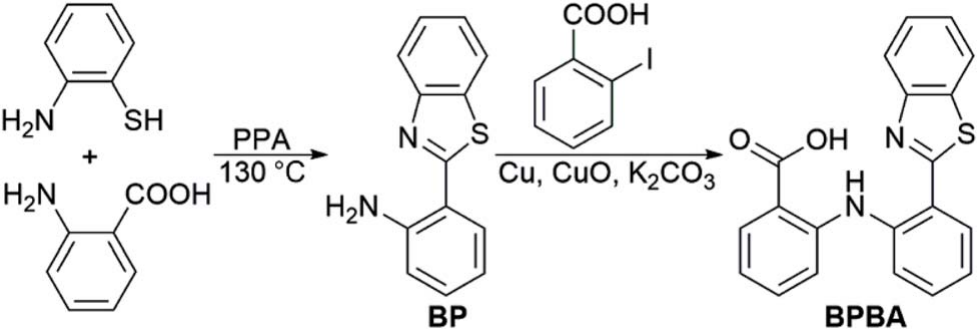
Synthetic route to BPBA.

The synthesis and characterization of BPBA are shown in Scheme 1 and Fig. S1.† The synthetic intermediate BP was synthesized by reacting 2-aminobenzoic acid with 2-aminothiophenol in polyphosphoric acid (PPA) as reported in the literature.^36^ BPBA was prepared by a modified literature method,^37^ which produced a yellow solid with a yield of 40%. BPBA is soluble in acetonitrile, methanol, and dimethyl sulfoxide (DMSO), but is insoluble in water. In the acetonitrile solution, two absorption peaks around 285 and 380 nm were detected by UV-vis spectroscopy, representing the existence of the benzene ring and the whole BPBA, respectively (Fig. S2A†). The maximum emission peak of BPBA is at 505 nm (*λ*_ex_ = 380 nm) in the emission spectrum (Fig. S2B†).

The blood–brain barrier (BBB) is the main obstruction for developing anti-AD agents.^38^ The BBB-penetrating ability of BPBA was firstly evaluated on the basis of Lipinski's “rule of five”, which predicts that a compound would cross the BBB when logBB is larger than 0.3.^39^ The calculated logBB of BPBA is 0.18 (Table S1†), and all other indices meet Lipinski's rule except Clog *P*, implying that it may potentially cross the BBB. The log *P*_octanol/water_ partition coefficient was further determined by the shaking flask method and UV spectroscopy. The lipophilicity (log *P*_o/w_) was calculated to be 1.24 ± 0.08 (Table S2†), suggesting that BPBA could penetrate the BBB.

### 2.2. Inhibition of Aβ aggregation

**Fig. 2:**
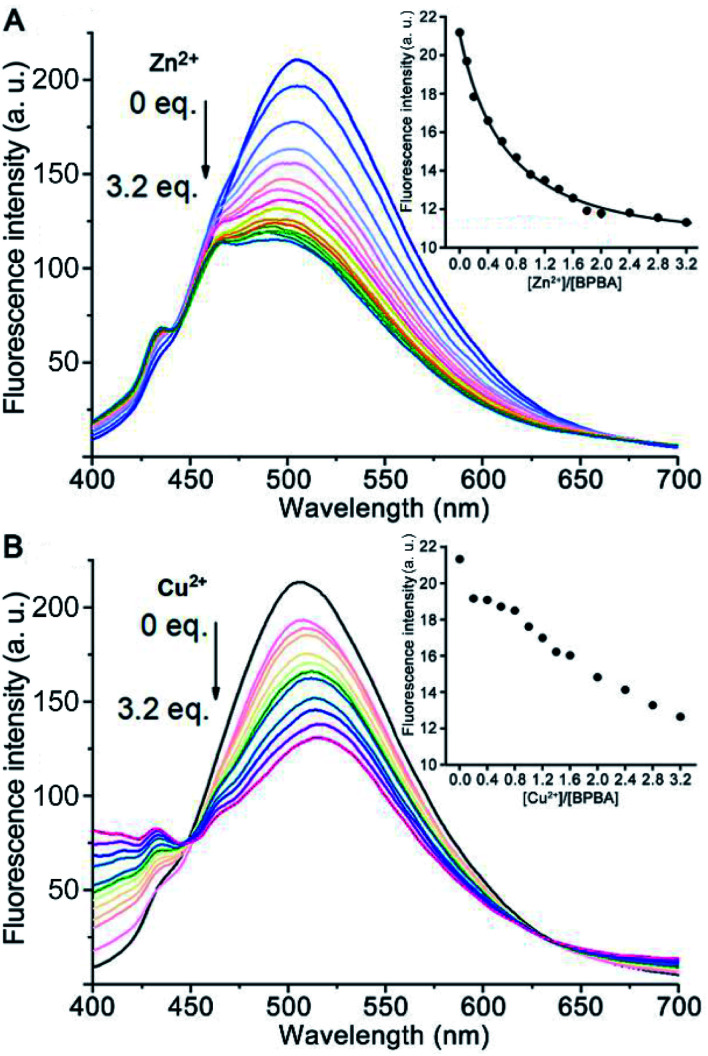
Fluorescence spectra of BPBA (20 μM) upon addition of increasing concentrations of Zn^2+^ (A) or Cu^2+^ (B) in the buffer (20 mM Tris–HCl, 150 mM NaCl, 4‰ v/v DMSO, and pH 7.4). Inset shows the emission intensity of BPBA at 505 nm *versus* different [Zn^2+^ or Cu^2+^]/[BPBA] ratios.

Excessive Zn^2+^ is associated with the generation of Aβ aggregates, and Cu^2+^ may lead to the aggregation of Aβ and production of reactive oxygen species (ROS),^40,41^ which would result in damage to neurons and synapses in AD patients.^42,43^ Since the O, N, and S atoms in BPBA could coordinate to metal ions, the chelating ability of BPBA to Zn^2+^ or Cu^2+^ was investigated by fluorescence titration in Tris–HCl buffer. As shown in Fig. 2, with the addition of Zn^2+^ or Cu^2+^, the maximum fluorescence intensity of BPBA decreased gradually and approached equilibrium when the ratio of [Zn^2+^]/[BPBA] reached 2.0, whereas the fluorescence intensity kept decreasing even when the ratio of [Cu^2+^]/[BPBA] reached 3.2. The results indicate that BPBA can bind to Zn^2+^ or Cu^2+^. However, since BPBA contains at least 4 coordination atoms and could form different chelates with Zn^2+^ or Cu^2+^, that is, the product is not unique, it is hard to identify these species in a complex mixture. Thus we only tentatively obtained an apparent binding constant of BPBA to Zn^2+^ at 1 : 1 stoichiometry, which was calculated to be 0.254 μM^−1^ according to the reported method.^44,45^ The chelation of BPBA to Zn^2+^ or Cu^2+^ was further confirmed by the HR-MS spectra in the Tris–HCl buffer, where the chelate cations of Zn^2+^ or Cu^2+^ with BPBA were observed (Fig. S3†).

**Fig. 3:**
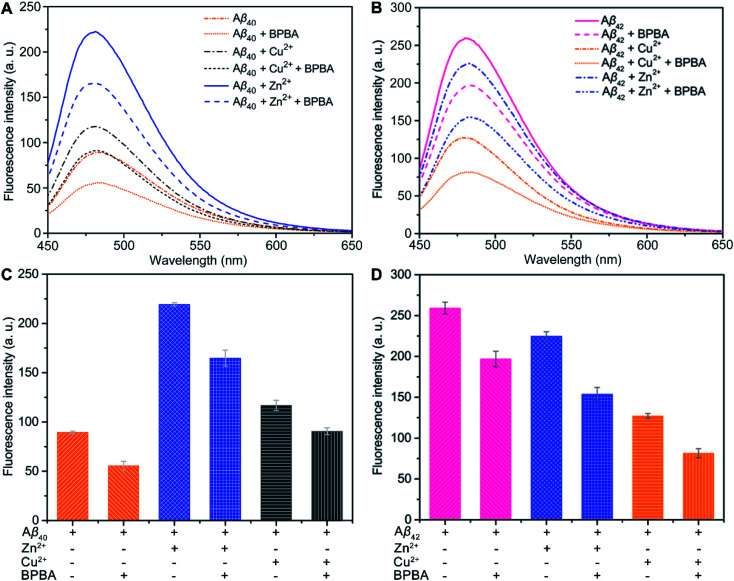
ThT fluorescence intensity (*λ*_ex_ = 415 nm and *λ*_em_ = 485 nm) of Aβ_40_ (A and C) and Aβ_42_ (B and D) solutions in the absence and presence of Zn^2+^ or Cu^2+^ after incubation with or without BPBA at 37 °C and pH 7.4 for 24 h ([Aβ] : [metal ions] : [BPBA] = 1 : 1 : 2 and [Aβ] = 20 μM).

The neurotoxicity of Aβ aggregates largely originates from the β-sheet conformers.^46^ The inhibition effect of BPBA on the β-sheet formation of Aβ was studied by the ThT assay, which is widely used to detect the content of β-sheets in Aβ aggregates.^47^ As presented in Fig. 3A and C, the fluorescence intensity increased obviously when Aβ_40_ was incubated with Zn^2+^ or Cu^2+^ as compared with Aβ_40_ alone, especially for Zn^2+^, suggesting that these metal cations can promote the formation of β-sheet aggregates and the effect of Zn^2+^ is greater than that of Cu^2+^. The fluorescence intensity of Aβ_40_ decreased when BPBA was added into the solution of Aβ_40_, Zn^2+^–, or Cu^2+^–Aβ_40_ aggregates, indicating that BPBA can inhibit the self- and metal-induced Aβ_40_ aggregation. Interestingly, the effect of Zn^2+^ or Cu^2+^ on Aβ_42_ is different from that on Aβ_40_. As shown in Fig. 3B and D, compared with the fluorescence intensity of Aβ_42_, in the presence of Zn^2+^ or Cu^2+^, the fluorescence intensity decreased apparently. This is consistent with the findings reported by Mirica, *et al.*, that is, Cu^2+^ stabilizes soluble Aβ_42_ oligomers, and Zn^2+^ leads to the formation of insoluble amorphous, non-fibrillar aggregates.^48^ Therefore, the aggregates of Aβ_42_ in the presence of Cu^2+^ are mainly formed by the self-aggregation of β-sheet conformers rather than by the induction of metal ions. Comfortingly, BPBA can also inhibit the self-aggregation of Aβ_42_ (Fig. S4†). The association constant of BPBA to Aβ_42_ was calculated to be 8.727 ± 4.023 μM^−1^. Although BPBA contains a ThT core, it did not interfere with the fluorescence of ThT in the presence of Aβ_42_ (Fig. S5†).

### 2.3. Morphological alteration of Aβ

**Fig. 4:**
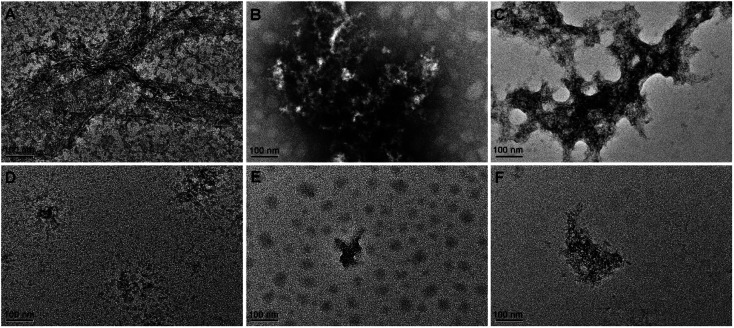
TEM images of Aβ_42_ in the absence or presence of Zn^2+^, Cu^2+^, and BPBA after incubation at 37 °C and pH 7.4 for 24 h. (A) Aβ_42_; (B) Aβ_42_ + Cu^2+^; (C) Aβ_42_ + Zn^2+^; (D) Aβ_42_ + BPBA; (E) Aβ_42_ + Cu^2+^ + BPBA; (F) Aβ_42_ + Zn^2+^ + BPBA ([Aβ_42_] : [metal cation] : [BPBA] = 1 : 1 : 2 and [Aβ_42_] = 20 μM).

The morphology of Aβ_42_ and Zn^2+^- or Cu^2+^-induced Aβ_42_ aggregates in the absence or presence of BPBA was further visualized by transmission electron microscopy (TEM). As shown in Fig. 4, some fibrils were formed in the solution of Aβ_42_, and large amounts of mature aggregates were observed after the addition of Zn^2+^ or Cu^2+^ to the Aβ_42_ solution, which are consistent with the literature;^49^ however, in the presence of BPBA, the Aβ_42_-induced fibrils and Zn^2+^- or Cu^2+^-induced Aβ_42_ aggregates changed into granule-like species or short fragments. The morphological changes indicate that BPBA can effectively inhibit the self-formed Aβ_42_ fibrils and metal-induced Aβ_42_ aggregates. The chelation of BPBA with metal ions is the primary reason for the disaggregation of Cu^2+^– and Zn^2+^–Aβ_42_ aggregates.

### 2.4. Influence on the hydrophobicity of Aβ

**Fig. 5:**
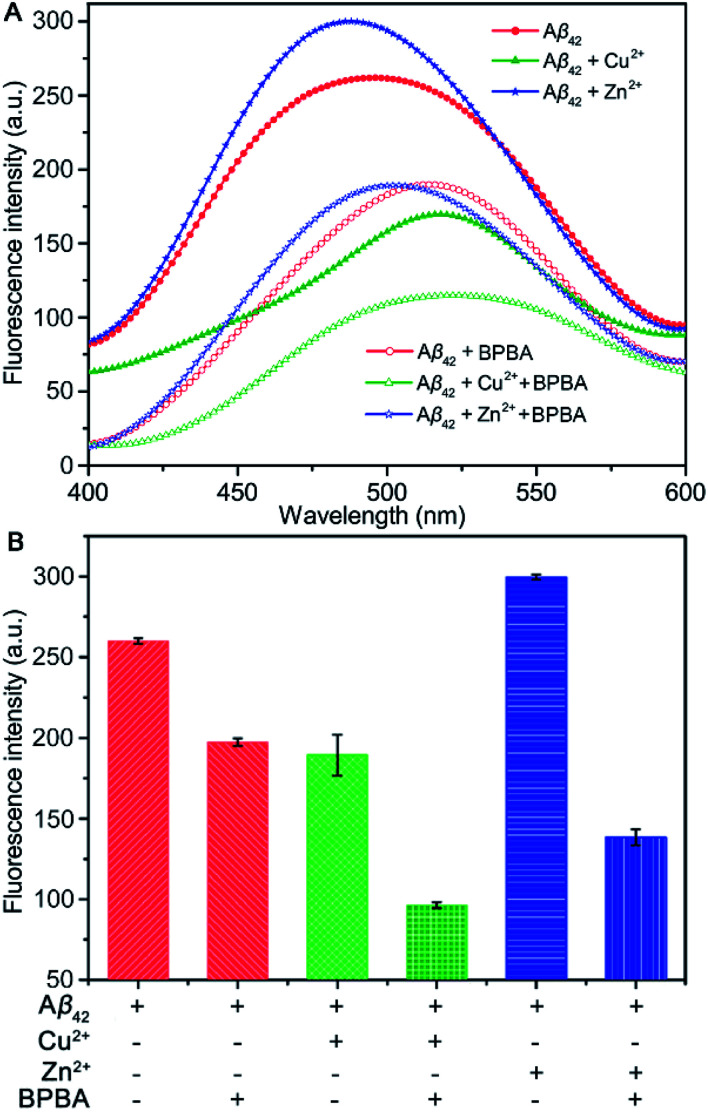
Fluorescence spectra (A) and fluorescence intensity (B) of ANS in Aβ_42_ solutions in the absence and presence of Zn^2+^ or Cu^2+^ after incubation with or without BPBA at 37 °C and pH 7.4 for 24 h ([Aβ] : [metal ions] : [BPBA] = 1 : 1 : 2, [Aβ] = 20 μM; *λ*_ex_ = 350 nm and *λ*_em_ = 400–600 nm).

Aβ fibrils are highly hydrophobic, which can be detected by using the fluorescent probe 8-aniline-1-naphthalenesulfonic acid (ANS).^50^ The fluorescence of ANS is enhanced significantly after binding to the hydrophobic structure on the surface of proteins.^51^ Therefore, the influence of BPBA on the hydrophobicity of Aβ can be reflected by the fluorescence changes of ANS. As shown in Fig. 5, the fluorescence intensity of ANS decreased and the maximum emission wavelength red-shifted once Cu^2+^ was added to the solution of Aβ_42_, suggesting that Cu^2+^ reduced the number of surface hydrophobic structures or the hydrophobicity of Aβ_42_ fibrils. In contrast, the fluorescence intensity of ANS increased after Zn^2+^ was incubated with Aβ_42_, indicating that Zn^2+^ increased the hydrophobicity of Aβ_42_ fibrils, and its impact on the hydrophobicity is different from that of Cu^2+^. Once BPBA was added into the above solutions, the fluorescence intensity of ANS decreased significantly, suggesting that BPBA has a remarkable inhibitory effect on the surface hydrophobic structures of self- and metal-induced Aβ_42_ aggregates. These results verify that BPBA can reduce the hydrophobicity or increase the hydrophilicity of Aβ_42_ aggregates. The fluorescence interference of BPBA with ANS is negligible on this occasion (Fig. S6†).

### 2.5. Effect of BPBA on nerve cells and Aβ toxicity

**Fig. 6:**
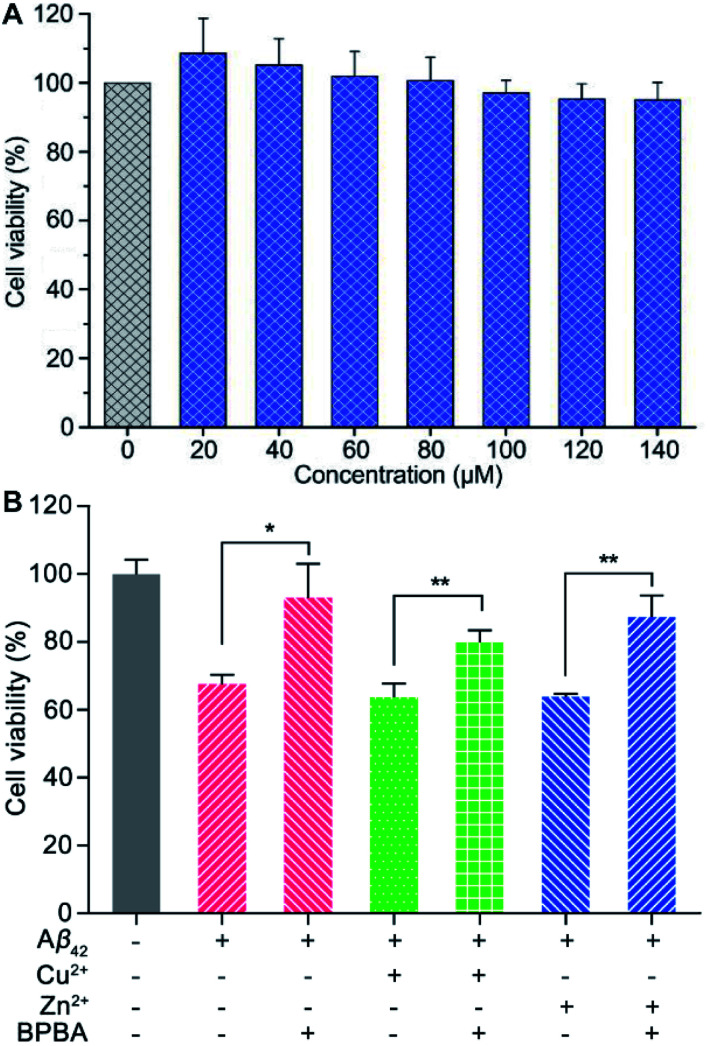
Viability of N2a cells after treatment with different concentrations of BPBA for 24 h (A), and viability of PC12 cells in the presence of Aβ_42_, Cu^2+^–Aβ_42_, and Zn^2+^–Aβ_42_ without or with BPBA after incubation for 24 h (B) ([Aβ_42_] = [Zn^2+^] = [Cu^2+^] = 10 μM, [BPBA] = 20 μM; **P < 0.001 and *P < 0.01).

Before assessing the effect of BPBA on the Aβ toxicity to neuron cells, we first examined the possible neurotoxicity of BPBA. Mouse neuroblastoma N2a (or Neuro-2a) cells are often used to study the pathological mechanism of AD.^52^ Therefore, the survival of N2a cells after incubation with BPBA for 24 h was tested by the MTT assay. As shown in Fig. 6A, the survival rate of N2a cells is around 100% even when the concentration of BPBA reached 140 μM, indicating that it is almost non-toxic to the neuron cells. The extremely low neurotoxicity suggests that BPBA *per se* is safe for neuron cells in the following experiments.

The neurotoxicity of Aβ_42_, Cu^2+^– and Zn^2+^–Aβ_42_ aggregates in the absence and presence of BPBA toward neuronal model cell PC12 from rat pheochromocytoma was tested using the MTT assay. As shown in Fig. 6B, Aβ_42_, Cu^2+^– and Zn^2+^–Aβ_42_ aggregates were markedly toxic to the PC12 cells, with the cell viability declining by more than 30%. The toxicity of Aβ_42_, Cu^2+^– and Zn^2+^–Aβ_42_ aggregates was significantly attenuated in the presence of BPBA, with the cell viability rising to more than 80%. The results show that BPBA can suppress the neurotoxicity of Aβ_42_, Cu^2+^– and Zn^2+^–Aβ_42_ aggregates and enhance the viability of neuron cells.

### 2.6. Inhibition on inflammasome

The NLRP3 inflammasome is an intracellular complex that activates caspase-1, which processes the IL-1β precursors into active molecules, and the mature IL-1β is released to the extracellular fluid.^13^ Human acute monocytic leukemia THP-1 cells are similar to the phenotype and functional characteristics of human primary monocytes/macrophages, therefore are commonly used as their model cells.^53^ THP-1 cells become mononuclear macrophages under the differentiation induced by phorbol ester (PMA). When the THP-1 macrophages were co-stimulated with lipopolysaccharide (LPS) and ATP, the NLRP3 inflammasome/caspase-1 was activated and a variety of cytokines like IL-1β were synthesized and released.^54^ Therefore, the effect of BPBA on the NLRP3 inflammasome was evaluated using the THP-1 macrophages.

**Fig. 7:**
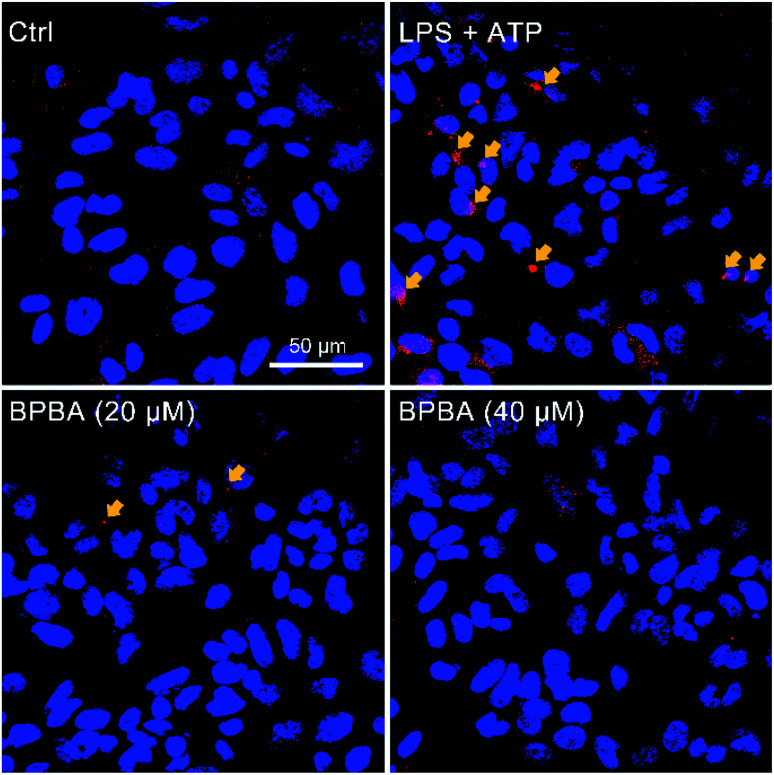
Immunofluorescence images of THP-1 macrophages before and after stimulation with LPS (1 μg mL^−1^) plus ATP (5 mM) and subsequent treatment with BPBA. Red, ASC specks; blue, DAPI-stained nuclei.

The NLRP3 inflammasome requires the adapter protein ASC to activate caspase-1. After inflammasome activation, ASC assembles into a large protein complex called “speck”, which can be detected by immunocytochemistry as the size reaches around 1 μm. Therefore, the formation of ASC specks is regarded as a simple upstream indicator of inflammasome activation.^25,55^ As shown in Fig. 7, red ASC specks were formed in the cytoplasm of THP-1 macrophages after stimulation with LPS and ATP; when the stimulated cells were treated with BPBA subsequently, the formation of ASC specks was dramatically inhibited, suggesting that BPBA can inhibit the activation of the NLRP3 inflammasome. Theoretically, the inhibition of BPBA on ASC specks should depend on its concentration; however, a quantitative relationship is unavailable because the exact position and amount of ASC specks are difficult to determine. According to the inhibition effect, 20 μM BPBA seems to be an appropriate concentration for the inhibition.

**Fig. 8:**
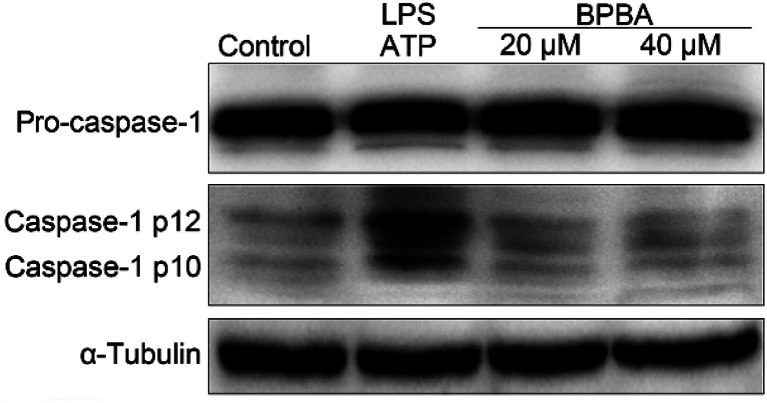
Expressions of pro-caspase-1 and caspase-1 in the LPS–ATP-activated THP-1 macrophages before and after the treatment with BPBA determined by western blotting.

As a result of ASC decline, the activation of caspase-1 was inhibited accordingly. As shown in Fig. 8, caspase-1 mainly exists as inactive pro-caspase-1 with or without stimulation; the activated caspase-1 p12 and caspase-1 p10 only increased after THP-1 macrophages were co-stimulated with LPS and ATP. However, in the presence of BPBA, the expression of activated caspase-1 decreased to the unstimulated level.

**Fig. 9:**
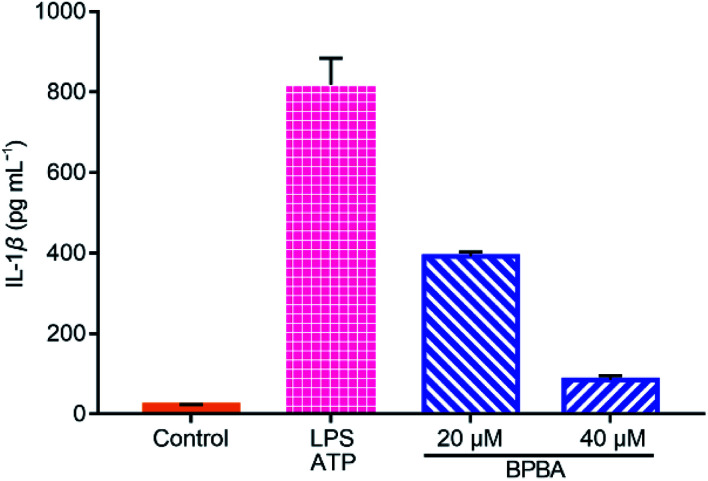
Levels of IL-1β in the LPS-ATP-activated THP-1 macrophages before and after the treatment with BPBA determined by ELISA.

IL-1β is a main inducer of inflammation and one of the main mediators of innate immune response, which is produced as an inactive precursor (pro-IL-1β) that requires cleavage by caspase-1 for activation and secretion.^56^ Its level is elevated in the brain of AD patients and is associated with the progression and onset of AD.^20^ As shown in Fig. 9, a large amount of IL-1β was secreted when THP-1 macrophages were stimulated with LPS and ATP, whereas the secretion of IL-1β was dramatically suppressed by BPBA, hence manifesting that BPBA can inhibit the release of IL-1β. All these results suggest that BPBA could inhibit the activation of the NLRP3 inflammasome and suppress the maturation and release of IL-1β – the final product of the inflammasome, thus predicting that BPBA could reduce the inflammatory response in AD patients.

### 2.7. Production of ROS and alleviation of paralysis in *C. elegans*

**Fig. 10:**
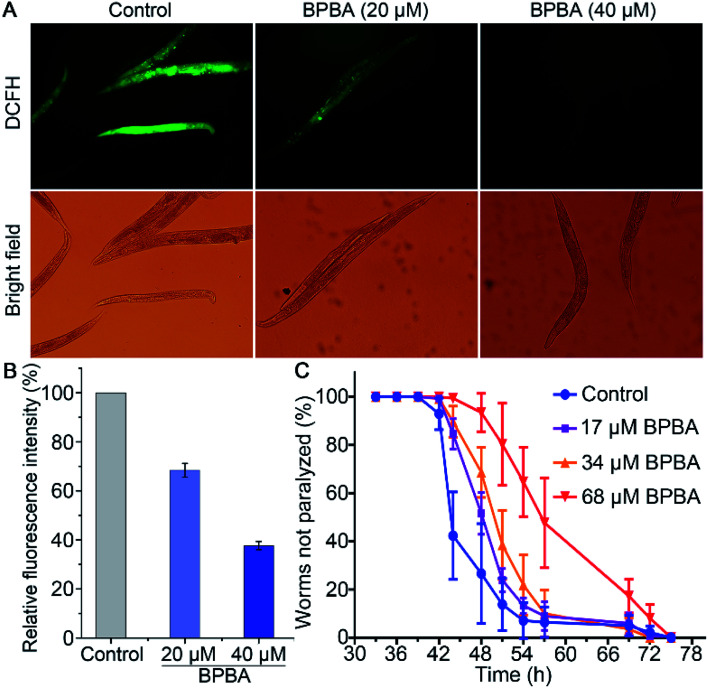
ROS level in CL4176 worms treated with BPBA 48 h after temperature rise measured with a H_2_DCF-DA fluorescence probe (A and B), and effect of BPBA on the paralysis of CL4176 worms at 25 °C (C). Results in (B) are expressed as relative fluorescence intensity normalized with protein concentration.

The transgenic *C. elegans* expressing Aβ gene in muscles or neurons is widely used as an *in vivo* model of AD to test the effect of compounds on Aβ aggregation and toxicity.^57^ Aβ plaques can induce ROS accumulation and oxidative stress, thus aggravating the pathological progress of AD ulteriorly.^58^ The changes of ROS in *C. elegans* CL4176 strains with or without BPBA were detected with a 2′,7′-dichlorodihydrofluorescein diacetate (DCFH-DA) probe. The ROS level was reflected by the DCF fluorescence that is positively dependent on the oxidization of DCFH by intracellular ROS.^59^ As shown in Fig. 10A, the green fluorescence intensity of BPBA-treated worms is significantly weakened as compared with that of the control, suggesting that BPBA can inhibit the production of ROS in the worms. The quantitative data of the fluorescence intensity are shown in Fig. 10B. The relative fluorescence intensity of the BPBA-treated group markedly decreased as compared to that of the control, indicating that BPBA can inhibit the level of ROS in *C. elegans*.

CL4176 specifically expresses the Aβ_42_ gene in the SMG-1 mRNA temperature induction system, which results in the time-dependent aggregation of Aβ and paralytic phenotypes.^60^ The activated SMG-1 system recognizes the Aβ gene and degrades it at 15 °C, causing the worms to produce a low level of Aβ and move as rollers; when the temperature is raised to 25 °C, the SMG-1 system is inactivated and Aβ aggregates are formed in the muscle cells, which paralyze the nematode gradually. The effects of BPBA on the Aβ-induced paralysis in CL4176 are shown in Fig. 10C. The BPBA-treated CL4176 nematodes were not paralyzed until 42 h after the temperature upshift; however, the paralysis rate of the untreated nematodes began to rise quickly thereafter, reaching more than 50% at 44 h. The paralysis rate of the worms treated with BPBA (68 μM) is only 7% at 48 h, while that of the control group is 73%. Evidently, BPBA can inhibit the Aβ-induced paralysis in transgenic CL4176 strains; in other words, it can downregulate the expression of the Aβ_42_ gene in CL4176 or alleviate the Aβ toxicity to neurons of *C. elegans*.

### 2.8. Reduction of Aβ and IL-1β in AD mice

**Fig. 11:**
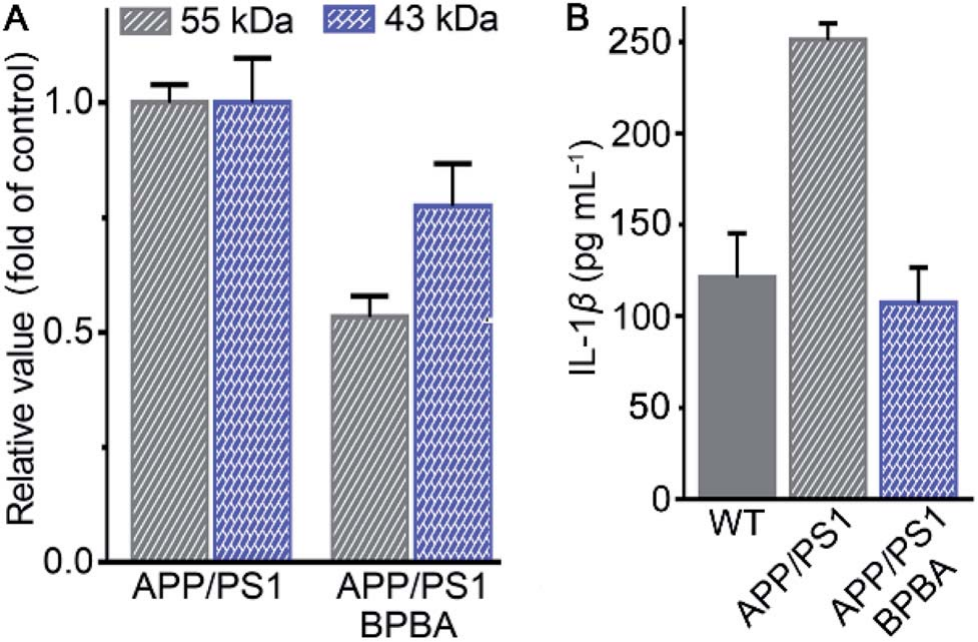
Content of different Aβ species relative to β-actin in the brain of APP/PS1 mice (A) and that of IL-1β in the brain of WT and APP/PS1 mice (B) after treatment with saline or BPBA (5 mg kg^−1^) every 3 days for 3 months.

The potential of BPBA to eliminate the pathogenic factors of AD was further verified in mice. APP/PS1 double-transgenic mice that produce elevated levels of Aβ by expressing human APP and PS1 mutants from 6 months of age were used as AD models.^61^ To select an appropriate dose for the assay, the acute toxicity of BPBA to wild type (WT) C57BL/6J mice was first evaluated. No obvious toxicity was observed at a dose of 10 mg kg^−1^ BPBA based on the mortality in 2 weeks (Fig. S7†). The APP/PS1 mice were treated with BPBA at a dose of 5 mg kg^−1^ every 3 days for 3 months. The brain tissues of the BPBA-treated and untreated AD mice were taken out and the expression of Aβ was analyzed by western blotting. As shown in Fig. 11A and S8,† the Aβ species with a molecular weight ≤55 kDa (oligomeric species) decreased significantly, thus confirming that BPBA can inhibit the formation of Aβ oligomers and plaques in the brain of AD mice. The results also imply that BPBA could pass through the BBB of the mice.

The effect of BPBA on pro-inflammatory IL-1β in the brain of AD mice was also investigated. As shown in Fig. 11B, the content of IL-1β in the brain of saline-treated wide-type (WT) and AD mice is 125 and 250 pg mL^−1^, respectively; however, that in the brain of BPBA-treated mice is 100 pg mL^−1^, which is even lower than that in the WT mice. The results demonstrate that BPBA can reduce the level of IL-1β *in vivo* and thereby douse the inflammatory responses. Since IL-1β is an immunomodulatory cytokine, the decrease of IL-1β production may further affect the innate immune cells in the brain.

### 2.9. Efficacy on APP/PS1 mice

**Fig. 12:**
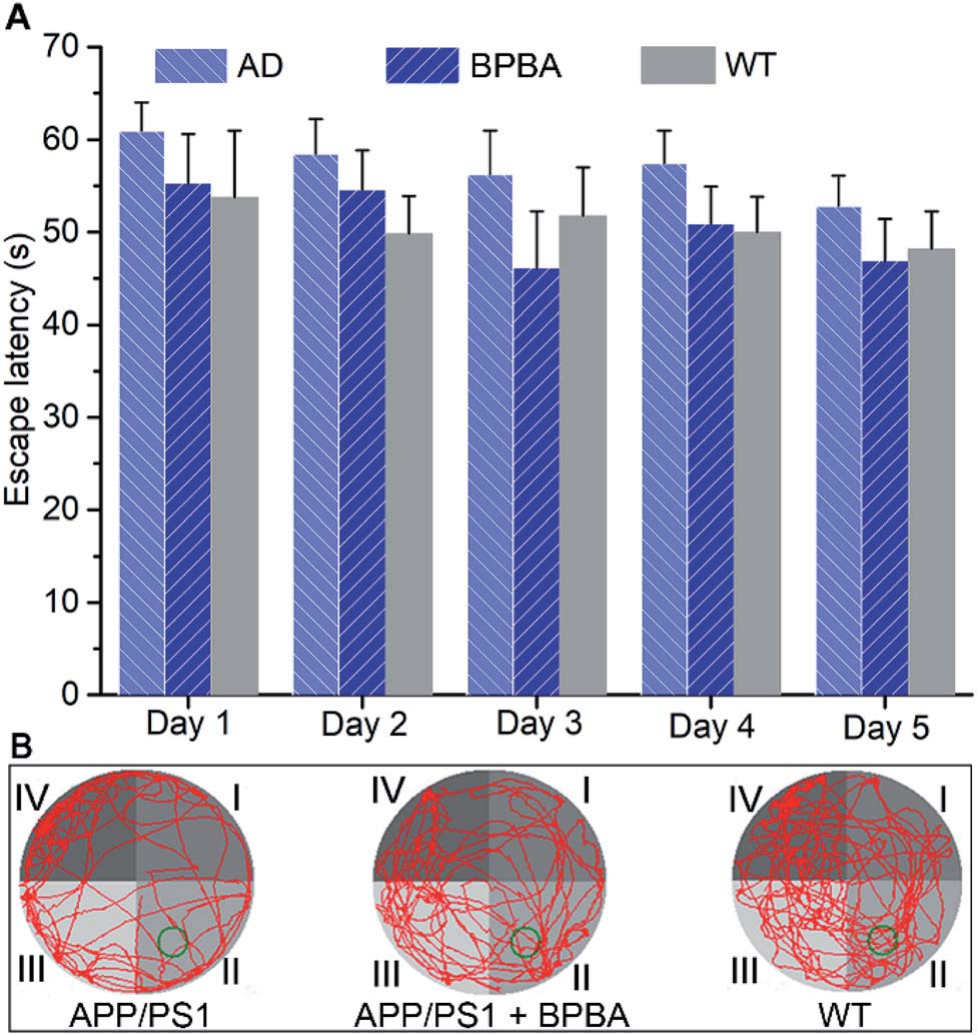
Effect of BPBA (5 mg kg^−1^) on the learning and cognitive abilities of APP/PS1 mice evaluated by the MWM test. (A) Escape latency time recorded daily during training trials, and data are presented as the mean ± S. D., and *n* = 6 each group; (B) swimming paths (red lines) of APP/PS1 mice with or without BPBA treatment on day 6 (green circle indicates the location of the survival platform).

Finally, the *in vivo* therapeutic effect of BPBA on the learning and cognitive abilities of APP/PS1 mice was assessed by the Morris water maze (MWM) test according to reported procedures.^61^ As shown in Fig. 12A, the time required for the BPBA-treated mice to find the survival platform is similar to that for the saline-treated WT mice, but is shorter than that for the AD control mice. On the 6th day, the survival platform was removed. The frequency of presence in the survival platform area (quadrant II) for the BPBA-treated mice increased within 60 s as compared with that for the saline-treated AD mice (Fig. 12B). Preliminary behavioral experiments show that BPBA can slightly alleviate the memory impairment of AD mice. The beneficial effect of BPBA on the memory of AD mice seems not so effective as expected, because the recovery of memory involves ceasing of neuron damage and regeneration of damaged neurons.^62^ BPBA can eliminate the pathogenic factors of neuron damage but cannot restore the damaged neurons. It is very possible that when the treatment began, the neuron damage had occurred, so the improvement of memory is not evident. This may be the reason why so many AD drug candidates failed in the clinic. Fortunately, the AD symptom did not get worse under the treatment of BPBA; in other words, BPBA can stop the progression of the disease, which is superior to most AD drugs.

## 3. Conclusion

Aβ aggregation is believed to be a key factor in the pathogenesis of AD; likewise, chronic neuroinflammation mediated by the activation of the NLRP3 inflammasome also plays a crucial role in the AD pathogenesis.^10,63^ Aβ deposits activate the NLRP3 inflammasome, leading to an overproduction of IL-1β and neuroinflammation. Therefore, dissociating Aβ aggregates not only directly reduces the Aβ-induced neurotoxicity to neurons, but also inhibits the activation of the NLRP3 inflammasome, curbs inflammatory responses, and decreases the release of neuro-destructive inflammatory cytokines.^17,31^ BPBA possesses the basic structural characters of both ThT and NSAID mefenamic acid, thus showing Aβ-targeting and anti-inflammatory abilities. Its function includes inhibiting Aβ aggregation, reducing ROS production, alleviating Aβ toxicity, deactivating the NLRP3 inflammasome, and restraining IL-1β release. *In vivo* studies on transgenic *C. elegans* show that BPBA can allay the Aβ-associated paralysis or Aβ toxicity to the neural system of *C. elegans*. Although BPBA cannot effectively recover the learning and cognitive abilities of AD mice, it can terminate the progression or deterioration of AD. The synergistic impact of BPBA on Aβ aggregation and neuroinflammation may bring about a new inspiration for the design of anti-AD drugs. Nevertheless, the exact mechanism of regulating the NLRP3 inflammasome as well as the interactions between NLRP3 inflammasome activation and other signaling pathways in AD remain to be clarified.

## Author contributions

T. Y. and L. Z. prepared the compounds and performed the experiments under the supervision of X .Y. W. and Z. J. G, Y. C. S. and Z. Z. Z. performed the western blotting and animal assays, S. X. J. and T. Y. analyzed the data and wrote the original draft, X. Y. W. edited the manuscript.

## Ethical statement

All animal experiments were performed in accord with the institutional animal use and care regulations approved by MARC and GDMLAC.

## Conflicts of interest

There are no conflicts to declare.

## Supplementary Material

SC-013-D1SC06071F-s001
