# Supplementary material for: Concurrent suppression of Aβ aggregation and NLRP3 inflammasome activation for treating Alzheimer's disease
Source: Chem Sci. 2022 Feb 21;13(10):2971–80. doi: 10.1039/d1sc06071f (PMC8905858; doi:10.1039/d1sc06071f)
Supplement: SC-013-D1SC06071F-s001 [file SC-013-D1SC06071F-s001.pdf]

Electronic Supplementary Material (ESI) for Chemical Science.
This journal is © The Royal Society of Chemistry 2022

Electronic Supplementary Information (ESI)

Concurrent suppression of A*β* aggregation and NLRP3 inflammasome activation for treating Alzheimer's disease

Tao Yang[#a], Lei Zhang[#a], Yicun Shang[a], Zhenzhu Zhu[a], Suxing Jin[b,c,*], Zijian Guo[c], and Xiaoyong Wang[a,*]

[a.] *State Key Laboratory of Pharmaceutical Biotechnology, School of Life Sciences, Nanjing University, Nanjing 210023, P. R. China. E-mail: boxwxy@nju.edu.cn; Fax: +86 25 89684549; Tel: +86 25 89684549*

[b.] *School of Food Science and Pharmaceutical Engineering, Nanjing Normal University, Nanjing 210023, P. R. China. E-mail: jinsuxing@njnu.edu.cn*

[c.] *State Key Laboratory of Coordination Chemistry, School of Chemistry and Chemical Engineering, Nanjing University, Nanjing 210023, P. R. China.*

[#] *These authors contributed equally.*

1. Supplementary Figures and Tables

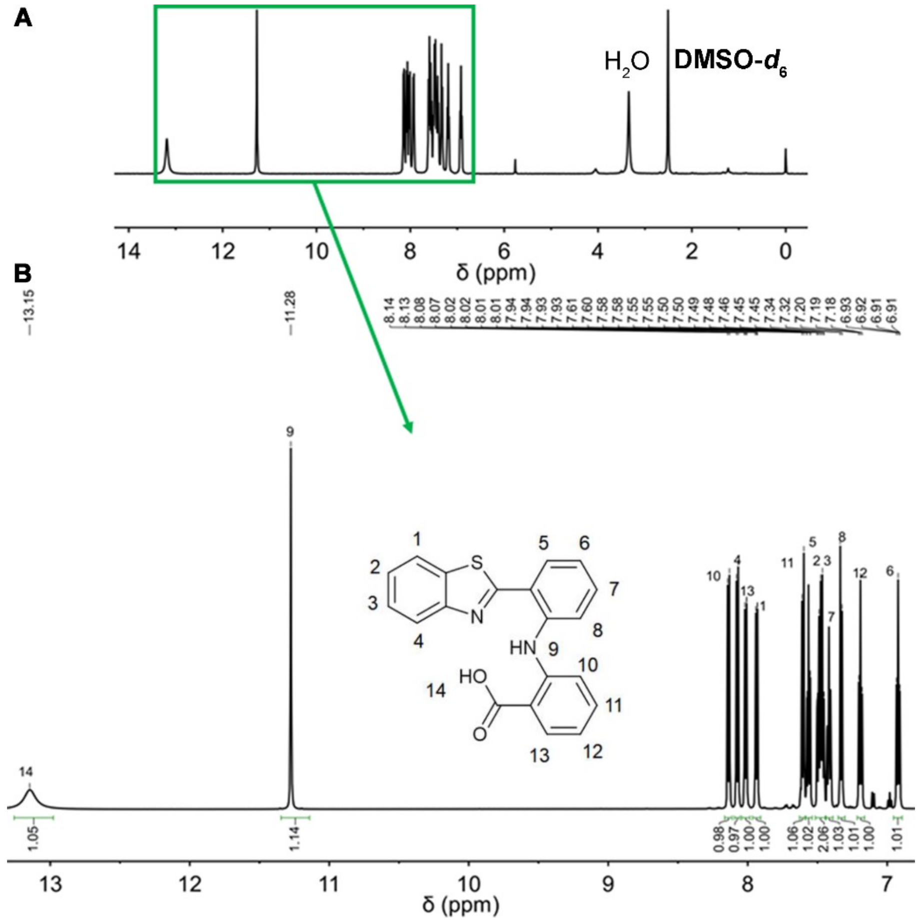

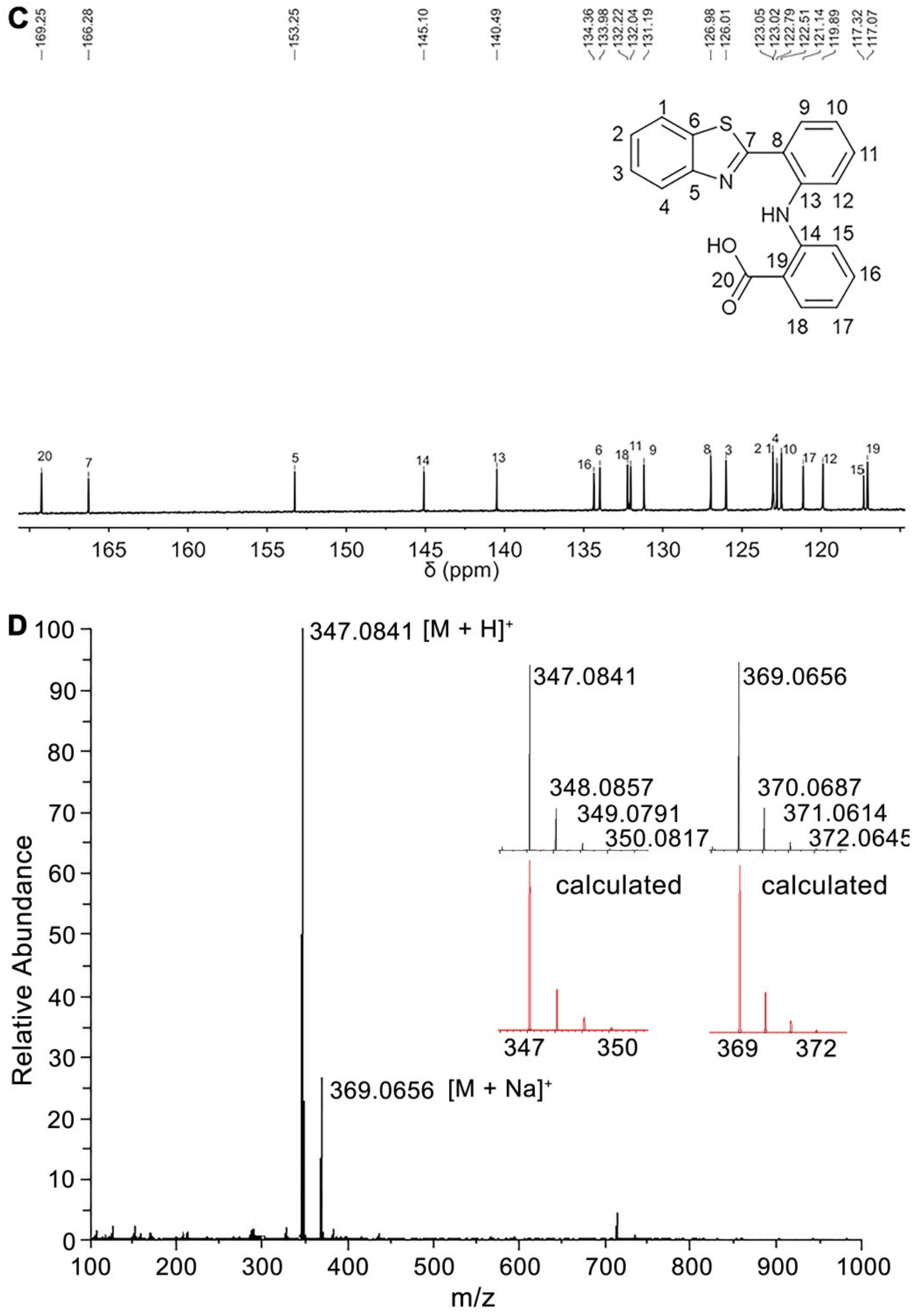

Fig. S1 (A, B) ^{1}H NMR (DMSO-d_6, 400 MHz, 298 K), (C) ^{12}C NMR (DMSO-d_6, 101 MHz, 298 K), and (D) HR-ESI-MS (CH_3OH) spectra of BPBA.

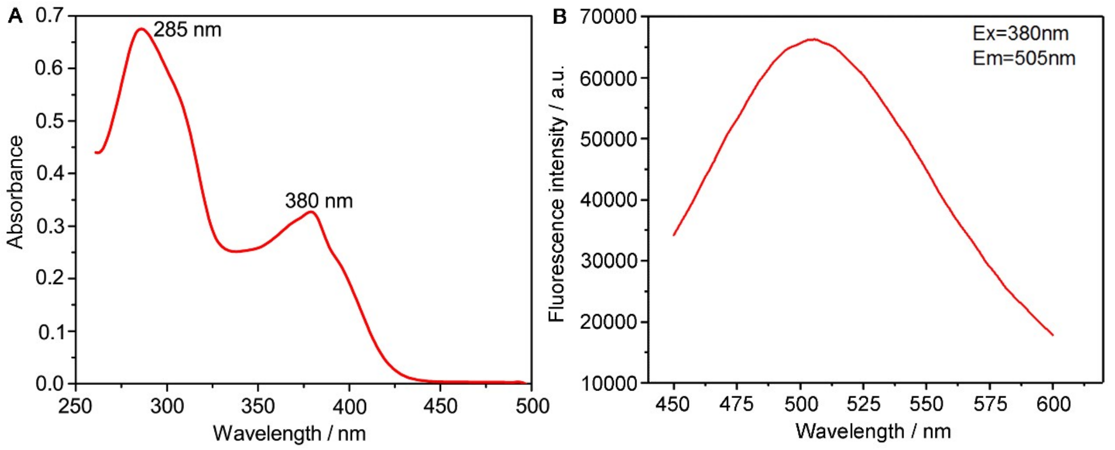

Fig. S2 UV-Vis absorption spectrum in acetonitrile (A) and fluorescence spectrum in buffer (20 mM Tris-HCl, 150 mM NaCl, pH 7.4, λ_{ex} = 380 nm) (B) of BPBA (40 μM).

Table S1. Molecular parameters recommended by Lipinski versus those of BPBA.

	MW	ClogP^a	HBA	HBD	PSA^a	logBB^b
Linpinski' rules	≤ 450	≤ 5	≤ 10	≤ 5	≤ 90	
BPBA	346	6.29	3	2	61.69	0.18

^{a} Clog$P$ and PSA are predicted by Discovery Studio 2.5 software (Accelrys); HBA, H-Bond Acceptors; HBD, H-Bond Donors; PSA, Polar Surface Area.

^{b} logBB = −0.0148 × PSA + 0.152 × Clog$P$ + 0.139; compounds with logBB > 0.3 are able to cross the BBB readily, with logBB < −0.1 are only poorly distributed to the brain.

Table S2. UV absorbance of BPBA at 372 nm and corresponding lipophilicity (log$P_{o/w}$) in phosphate buffer and 1-octanol at different concentrations.

Compound	Concentration (μM)	Ab_{buffer}		$Ab_{octanol}$	log$P_{o/w}$	
		Before shaking	After shaking			
BPBA	50	0.1753	0.0103	0.1650	1.2046	1.24 ± 0.08
	75	0.2380	0.0106	0.2274	1.3314	
	100	0.2664	0.0167	0.2497	1.1746	

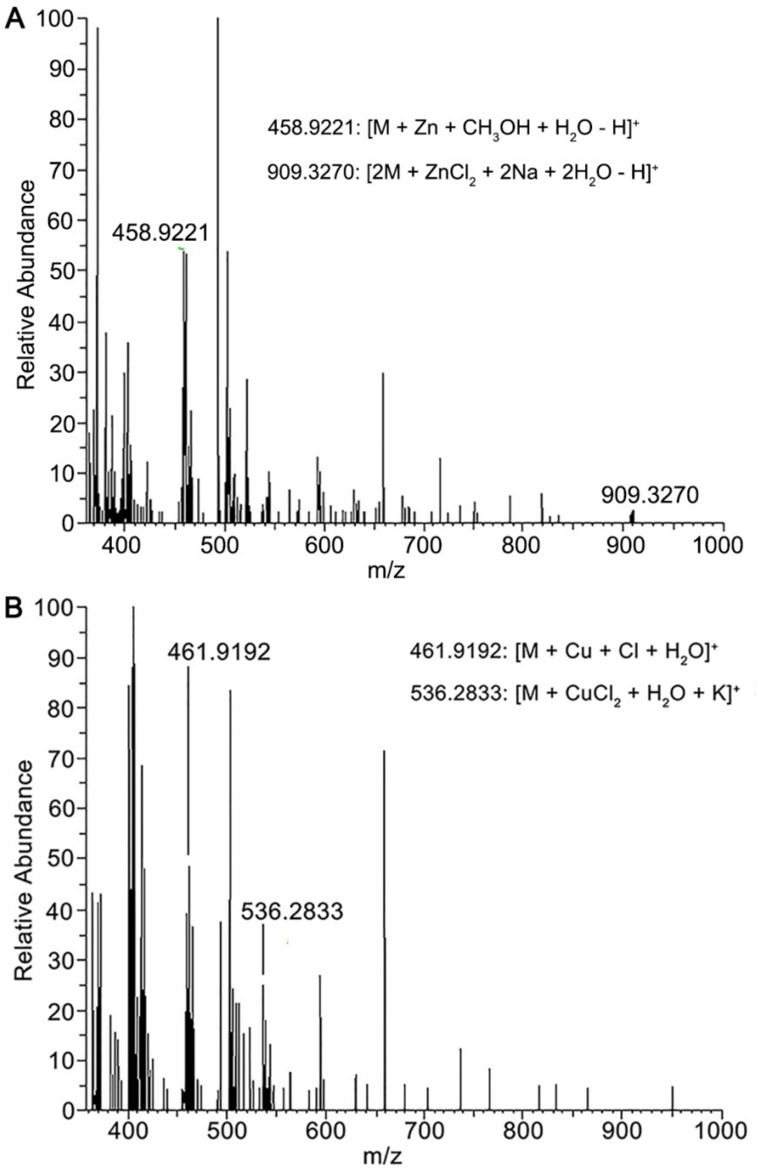

Fig. S3 HR-MS spectra of BPBA (40 μM) with (A) Zn^{2+} or (B) Cu^{2+} (40 μM) in the buffer (20 mM Tris-HCl, 150 mM NaCl, pH 7.4).

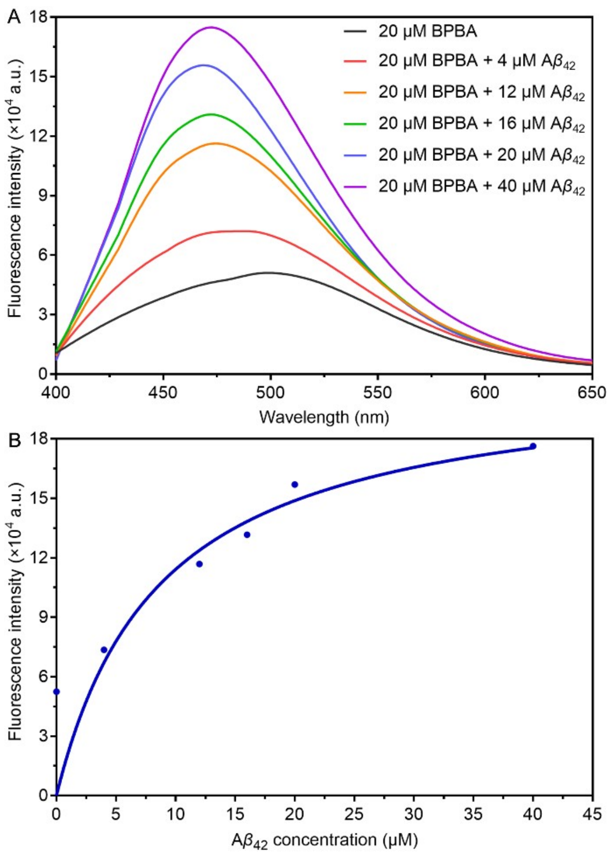

Fig. S4 Fluorescence spectra (A) and quantitative results of fluorescence intensity (B) of BPBA (20 μM) in the absence and presence of different concentrations of Aβ_{42} in the buffer (20 mM Tris-HCl, 150 mM NaCl, pH 7.4).

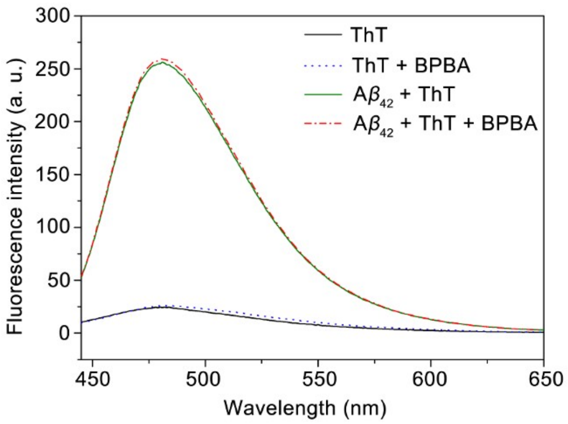

Fig. S5 Fluorescence spectra of ThT (20 μM) with or without Aβ_{42} (20 μM) in the absence or presence of BPBA (40 μM) in the buffer (20 mM Tris-HCl, 150 mM NaCl, pH 7.4).

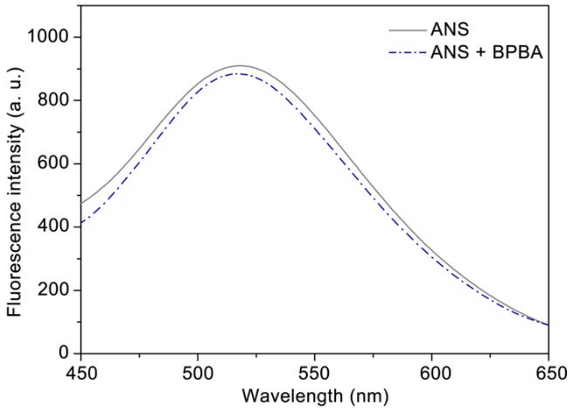

Fig. S6 Fluorescence spectra of ANS (20 μM) in the absence and presence of BPBA (40 μM) in the buffer (20 mM Tris-HCl, 150 mM NaCl, pH 7.4).

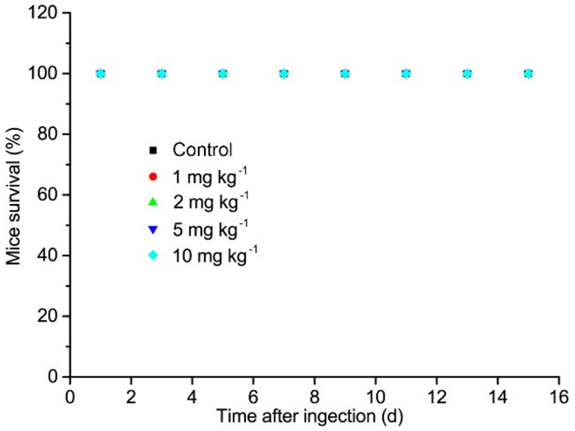

Fig. S7 Survival rate of C57BL/6J mice after injection of BPBA via the tail vein every other day at a dosage of 0, 1, 2, 5, and 10 mg kg^{-1}, respectively.

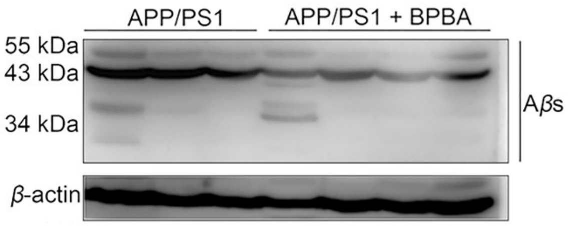

Fig. S8 A*β* species in the brain of APP/PS1 mice after treatment with saline or BPBA (5 mg kg^{-1}) every 3 days for 3 months determined by Western blotting.

2. Experimental section

2.1 Materials and methods

All the reagents and solvents were of analytical grade and used as received without further purification. 2-Aminothiophenol, 2-aminobenzoic acid, polyphosphoric acid

(PPA), 2-iodobenzoic acid, dichloromethane (DCM), petroleum ether (PE), copper oxide, potassium carbonate, copper, copper chloride and zinc chloride were purchased from Energy-Chemical (Shanghai, China). Human Aβ_{40} and Aβ_{42} were purchased from GL Biochem Ltd. (Shanghai, China). Stock solutions of Aβ_{40} and Aβ_{42} were prepared according to the literature method.[1] Thioflavin T (ThT), 3-(4,5-dimethylthiazol-2-yl)-2,5-diphenyltetrazolium bromide (MTT), 8-aniline-1-naphthalenesulfonic acid (ANS), phorbol ester (PMA), lipopolysaccharide (LPS), adenosine triphosphate (ATP), agar, cholesterol, yeast powder, peptone, 5-fluoro-2′-deoxy-b-uridine (FUdR), and 2,7-dichlorofluorescein diacetate (H_2DCF-DA) were purchased from Sigma-Aldrich Company. BPBA stock solution (10 mM) was prepared by dissolving the compound in dimethyl sulfoxide (DMSO). All solutions and buffers were prepared using Milli-Q water, and filtered through a 0.22 μm filter (Millipore) before use. Cell-lysis RIPA buffer and BCA protein assay kit were purchased from Beyotime Biotechnology (Shanghai, China). Anti-pro-caspase-1 +p10 +p12 (ab179515), β-actin (ab8227) antibodies and goat anti-rat IgG H&L (Alexa Fluor® 647, ab150159) secondary antibody were purchased from Abcam. 6E10 monoclonal antibody was purchased from Covance. The mouse N2a neuroblastoma cells and human acute monocytic leukemia THP-1 cells (THP-1) were purchased from American Type Culture Collection (ATCC).

Electrospray ionization mass spectra (ESI-MS) were determined on an LCQ spectrometer (Finnigan). ^{1}H- and ^{13}C NMR spectra were acquired on a Bruker DRX-400 and Bruker Avance III 400 spectrometers at 298 K. Fluorescence spectra were recorded on a HORIBA Fluoromax-4P fluorescence spectrometer. Transmission electron microscopic (TEM) images were taken with a transmission electron microscope (JEOL, JEM-2100 LaB6). The optical density (OD) of formazan was determined using a Tecan Sunrise ELISA Reader at 570 nm. *C. elegans* was observed by Stereo Microscope, Motic. Fluorescence photographs were recorded by fluorescent microscopy (Ningbo Sunny instruments Co. Ltd.). Western blotting was carried out on the Bio-Rad mini-PROTEAN tetra system and Bio-Rad Powerpack Universal. Images

were captured using a Chemiscope 3400 mini (Clinx science instrument co. Ltd). Data were expressed as means ± standard deviation (S. D.).

Transgenic CL4176 strains were obtained from the Caenorhabditis Genetics Center (University of Minnesota, Minneapolis, MN, USA). The worms were cultured on the standard Nematode Growth Media (NGM) plates spotted with *E. coli* strain OP50 grown in Luria Broth (LB) medium. The detailed experimental procedures were described elsewhere.[1]

C57BL/6J mice and APP/PS1 transgenic mice were purchased from the Model Animal Research Center of Nanjing University (MARC) and Guangdong Medical Laboratory Animal Center (GDMLAC), respectively. All animal experiments were performed in accord with the institutional animal use and care regulations approved by MARC and GDMLAC.

2.2 Synthesis of BPBA

The synthetic intermediates BP was synthesized by reacting 2-aminobenzoic acid with 2-aminothiophenol in PPA as reported in the literature.[2] BPBA was prepared using a published method with some modifications.[3] Specifically, copper oxide (0.1 g, 1.25 mmol), potassium carbonate (0.08 g, 0.58 mmol), and copper (0.2 g, 3.12 mmol) were added to the ethanol solution of 2-iodobenzoic acid (2.50 g, 10 mmol) and BP (4.50 g, 20 mmol). After stirring at 70 °C for 24 h, a suspension was obtained. The filtrate was collected after filtration and dried in vacuum to get crude product. The product was purified using column chromatography and a yellow solid product was obtained with a yield of 40%. ^{1}H NMR (DMSO-d_6, 400 MHz, δ, ppm): 13.19 (s, 1H, –COOH), 11.27 (s, 1H, –NH–), 8.15–8.13 (d, 1H, Ph), 8.08–8.06 (d, 1H, benzothiazole), 8.03–8.01(d, 1H, Ph), 7.94–7.92(d, 1H, benzothiazole), 7.61–7.54 (q, 2H, Ph), 7.51–7.40 (m, 3H, benzothiazole and Ph), 7.34–7.32(d, 1H, Ph), 7.21–7.17 (t, 1H, Ph), 6.94–6.90 (t, 1H, Ph). ^{13}C NMR (DMSO-d_6, 151 MHz, δ, ppm): 169.25, 166.28, 153.25, 145.10, 140.49, 134.36, 133.98, 132.22, 132.04, 131.19, 126.98, 126.01, 123.05, 123.02, 122.79, 122.51, 121.14, 119.89, 117.32, 117.07. HR-MS (m/z)

found (calcd) for $[C_{20}H_{14}N_2O_2S + H]^+$: 347.0841 (347.0854); $[C_{20}H_{14}N_2O_2S + Na]^+$: 369.0656 (369.0674).

2.3 Octanol/water partition coefficient (Log $P_{o/w}$)

The lipophilicity of BPBA was measured in an octanol/buffer system using the shaking flask method and UV spectroscopy. Solutions of BPBA (50, 75, 100 μM) containing DMSO (1%) were prepared in the PBS (pH 7.4) presaturated with octanol. Equal volumes (2.0 mL) of the solution and octanol presaturated with PBS were mixed and shaken at room temperature for 24 h and then separated into two phases by centrifugation. The concentration of the solute in the aqueous phase was determined by spectrophotometry (λ_{max} = 372 nm). According to the law of mass conservation, the compound concentration in the octanol phase and the lipo-hydro partition coefficient $P_{o/w}$ ($P_{o/w} = C_o/C_w = A_o/A_w$, where A stands for absorbance) were calculated. The log P values were calculated on the average of 3 independent measurements.

2.4 Spectral properties and fluorescence titration of BPBA

The UV-Vis absorption spectra of BPBA were determined in DMSO and the fluorescence spectra were determined by adding BPBA solution (40 μM) to 3 mL buffer (20 mM Tris-HCl, 150 mM NaCl, pH 7.4) in a cuvette. The fluorescence titration of BPBA (λ_{ex} = 505 nm) was carried out by adding aliquots of $ZnCl_2$ or $CuCl_2$ aqueous solution (5 mM, 1.2 μL) to 3 mL of BPBA solution (20 μM, 20 mM Tris-HCl, 150 mM NaCl, pH 7.4) in a cuvette.

The binding constant (K_d) of the complex between Zn^{2+} and BPBA was determined by direct fluorometric titration according to the reported method.[4,5] The fluorescence intensity data was fitted to Eqn (1), assuming a 1:1 stoichiometry between BPBA and Zn^{2+}. Nonlinear fitting of Log [Zn^{2+}] to Log $(F-F_{min})/(F_{max}-F)$ recorded as a function of [Zn^{2+}] gave the value of K_d. In the equation, F stands for the fluorescence intensities at [X] (X = Zn^{2+}), F_{min} and F_{max} are the fluorescence intensities with no addition of X and addition of X at 505 nm, respectively; n is the binding stoichiometry between BPBA and Zn^{2+}.

$$F = \frac{F_{min} K_d + F_{max} [X]^n}{K_d + [X]^n} \quad (1)$$

2.5 Inhibition on A*β* aggregation

Aβ_{40} and Aβ_{42} (20 μM) was incubated with or without $CuCl_2$ or $ZnCl_2$ (20 μM) in buffer solution (20 mM Tris-HCl/150 mM NaCl, 700 μL, pH 7.4) at 37 ºC for 5 min, respectively. BPBA (0.4 μL, 20 mM) or DMSO (2.8 μL, final concentration 4‰) was added and incubated at 37 ºC for 24 h. Each sample (200 μL) was transferred to a 96-well black plate (Corning Costar Corp) and ThT (0.8 μL, 5 mM) was added to each solution in the dark. The fluorescence intensity was recorded by a Varioskan Flash microplate reader (Thermo Scientific) (λ_{ex} = 415 nm, λ_{em} = 485 nm). Data were expressed as mean ± standard deviations (S. D.) of at least 3 independent experiments.

ThT was replaced by ANS, and the fluorescence intensity was recorded (λ_{ex} = 370 nm, λ_{em} = 400–600 nm) to assess the effect of BPBA on the hydrophobicity of A*β*.

2.6 Morphological changes of A*β*

TEM samples were prepared in the same way as described in the above ThT assay. An aliquot solution (10 μL) was spotted on the 300-mesh carbon-coated copper grids within 2 min at room temperature and excess sample was removed. Each grid was stained with uranyl acetate (1.5%, w/v) for 1 min and then washed with Milli-Q water. Uranyl acetate was blotted up and the grids were dried for 20 min at room temperature. The samples were examined on a JEOL JEM-2100 LaB6 (HR) transmission electron microscope.

2.7 Cytotoxicity and detoxification ability

N2a cells were cultivated overnight in DMEM supplemented with 10% heat-inactivated fetal bovine serum in an incubator under 5% CO_2 at 37 °C, and planted in 96-well plates with 6×10^3 cells per well. BPBA was dissolved in DMSO and diluted with culture medium to make the concentration of DMSO lower than 5‰. The stock solution of BPBA was diluted with a complete medium and then added into the wells. After the cells were incubated for 24 h, MTT (20 μL, 5 mg mL^{-1}) was added to each

well and incubated for 4 h. The medium was removed and DMSO (150 μL) was added to the cells. The absorbance of the solution (OD) was recorded on an ELISA plate reader at 570 nm. Each test was performed in triplicate. The cell viability (%) was calculated using the following equation:

$$\text{Cell viability (\%)} = [(OD_{sample} - OD_{blank})/(OD_{control} - OD_{blank})] \times 100\%$$

PC12 cells were seeded in a 96-well flat bottomed microplate at 5.5 × 10^3 cells per well and incubated at 37 °C in a humidified atmosphere with 5% CO_2. Aβ_{42}, Zn^{2+} or Cu^{2+} (10 μM) was added into the cells at 37 °C for 15 min, followed by addition of BPBA (20 μM, final DMSO concentration 4‰). After incubation at 37 °C for 24 h, MTT (40 μL, 2.5 mg mL^{-1}) was added to each sample solution. The samples were re-incubated for 4 h at 37 °C in a humidified atmosphere with 5% CO_2. The supernatants were removed and the formazan crystals were dissolved in DMSO (150 μL). The absorbance at 490 nm was determined using a Varioskan Flash microplate reader.

2.8 Effect on inflammasome

THP-1 cells were planted into a 6-cm plate (10% fetal bovine serum in RPMI 1640 with 2-mercaptoethanol) and allowed to grow overnight. PMA was added to the THP-1 cells and co-incubated for 24 h; the medium was replaced with serum-free medium to starve the cells for 12 h. The cells were treated with BPBA for 4 h, stimulated with LPS (1 μg mL^{-1}) for 4 h, and further stimulated with adenosine triphosphate (ATP, 5 mM) for 1 h to induce the activation of NLRP3 inflammasome. The expression of activated caspase-1 protein in cells was detected by Western blot. ELISA was performed on the supernatants against IL-1β according to the manufacturer's protocol.

Proteins in lysates were separated on 12% SDS-PAGE gel and transferred to a PVDF membrane. The membrane was blocked for 1 h at room temperature with 5% fat-free milk powder and probed with primary antibodies anti-pro-Caspase-1 +p10 +p12 (ab179515) (1:1000) and α-tubulin (1:5000) at 4 °C overnight. The appropriate secondary antibody was incubated with HRP-labeled goat anti-rabbit (1:10000) for 1 h

at room temperature. After incubation, the antibodies were washed in TBS-T buffer and the bands were visualized with SuperSignal (Millipore).

The formation of ASC specks in THP-1 macrophages was evaluated by immunocytochemistry. THP-1 cells were seeded in 20 mm glass bottom cell culture dishes (Nest) and incubated at 37 °C for 18 h before adding compound. After treatment as described above, the cells were washed with PBS and fixed with 4% paraformaldehyde for 10 min. The cells were washed by cold PBS for 5 min, blocked with 0.5% BSA dissolved in PBS for 15 min at room temperature. The cells were then incubated with the anti-TMS1/ASC primary antibody (ab155970, Abcam) at 4 °C overnight. After incubation, the cells were washed twice with PBS and incubated with specific secondary antibody for 1 h at room temperature. Finally, the cells were washed 3 times with PBS and observed under ZEISS Laser Scanning Microscope (LSM 710).

2.9 Analysis of transgenic *C. elegans*

CL4176 nematodes were transferred onto NGM plates spread with *E. coli* OP50 mixed with or without BPBA for laying eggs at 15 °C. The gravid adults were removed and the progenies were allowed to reach the third larval (L3); some worms were then transferred onto new plates spotted with FUdR (50 mL, 0.5 mM). The temperature was upshifted to 25 °C, which lead to the increase of $A\beta_{42}$ production. The paralyzed worms were counted every 3 h after 20 h of the temperature upshift until all worms on each plate were paralyzed.

2.10 Measurement of ROS in *C. elegans*

Intracellular ROS in CL4176 strains was measured using H_2DCF-DA. Freshly laid CL4176 eggs (100 eggs per plate) were transferred to NGM plates containing BPBA (20, 40 μM) and incubated at 15 °C. The temperature was raised to 25 °C for 48 h. The worms were collected by M9 buffer, washed twice with PBS to remove *E. coli* OP50, transferred to a 96-well plate (Costar) with 200 mL of PBS containing Tween 20 (0.01%), and H_2DCF-DA (100 μM) was added. The fluorescence intensity (λ_{ex} = 485 nm, λ_{em} = 530 nm) was quantified by a microplate reader after 6 h. The collected worms

were destained by transferring to fresh NGM plates for 2 h, mounted on slides and observed with a microscope (DM4000B; Leica, Germany) equipped with a digital camera. Data were represented as mean ± S.D., n = 3.

2.11 In vivo acute toxicity

C57BL/6J mice (8 weeks old, male) were randomly divided into 4 experimental groups and one control group (5~6 mice in each). Experimental mice were administered with 1, 2, 5, and 10 mg kg^{-1} of BPBA via tail vein, while the control mice were administered with saline every other day for 2 weeks. The changes in the mortality of mice in each group were recorded every other day during the treatment.

2.12 Analysis on in vivo A*β* and IL-1*β*

APP/PS1 mice were intravenously administered with BPBA (5 mg kg^{-1}) or saline every 3 days for 3 months. The brains of WT, APP/PS1 mice and BPBA-treated mice were collected and homogenized in RIPA buffer (10 mM Tris–HCl, pH 7.5, 50 mM NaCl, 0.5% NP-40, 4 mM EDTA, 0.1% SDS, 0.5% sodium deoxycholate and protease inhibitor cocktail) on ice for 15 min and centrifuged at 16,000 g and 4 °C for 30 min. The supernatant of brain lysates was dissolved in loading buffer and boiled at 95 °C for 15 min, which was then analyzed by Western blotting as described above. The brains of WT, APP/PS1 and BPBA-treated mice were collected with saline, homogenized on the ice and centrifuged at 16,000 g and 4°C for 15 min. The supernatant was collected and total protein in it was quantified using BCA protein assay kit (Beyotime). ELISA was performed on the supernatant against IL-1*β* according to the manufacturer's protocol.

2.13 Morris water maze test

APP/PS1 mice were randomly allocated into two groups (n = 6 for each group) when they were 6 months old, and intravenously administered with BPBA (5 mg Kg^{-1}) or saline every 3 days for 3 months. The mice were then examined by MWM test after 5 days of platform training.[6] To confirm the inhibition of BPBA on cognition and memory decline, age-matched WT mice (9-month-old, male) were also subjected to

behavioral test. The test was consisted of 4 platform trials per day for consecutive 5 days, and followed by a probe trial on the 6th day. In platform trials, the escape latency was measured. After training, the mice were tested to search for the platform in the MWM apparatus, where they were permitted to swim freely for 60 s. Data were recorded and analyzed through a video camera-based Ethovision System (Nodulus, the Netherlands).

Reference

[1] T. Yang, Z. Z. Zhu, E. M. Yin, Y. Q. Wang, C. L. Zhang, H. Yuan, H. M. Zhang, S. X. Jin, Z. J. Guo, and X. Y. Wang, *Chem. Sci.*, 2019, **10**, 10149–10158.

[2] J. Y. Zhang, and W. Guo, *Chem. Commun.*, 2014, **50**, 4214–4217.

[3] S. L. Chandak, A. S. Bansode P. R. Murumkar, M. G. Shinde, and K. G. Bothara, *Med. Chem. Res.*, 2013, **22**, 3510–3517.

[4] M. Baruah, W. W. Qin, R. A. L. Vallée, D. Beljonne, T. Rohand, W. Dehaen and N. Boens, *Org. Lett.*, 2005, **7**, 4377–4380.

[5] S. Liu, L. W. Zhang, X. Liu, *New J. Chem.*, 2013, **37**, 821–826.

[6] X. M. Zha, D. Lamba, L. L. Zhang, Y. H. Lou, C. X. Xu, D. Kang, L. Chen, Y. G. Xu, L. Y. Zhang, A. D. Simone, S. Samez, A. Pesaresi, J. Stojan, M. G. Lopez, J. Egea, V. Andrisano, and M. Bartolini, *J. Med. Chem.*, 2016, **59**, 114–131.
